# Aging Affects the Demands and Patterns in Active Control Under Different Sensory-Conflicted Conditions

**DOI:** 10.3389/fnagi.2021.742035

**Published:** 2021-11-05

**Authors:** Jing Hu, Jung Hung Chien

**Affiliations:** Department of Health & Rehabilitation Sciences, College of Allied Health Professions, University of Nebraska Medical Center, Omaha, NE, United States

**Keywords:** aging, gait, sensory, perturbation, active control

## Abstract

Most falls might be attributed to an unexpected perturbation such as a slip. It might be aggravated by the deterioration of the sensory system as people aged. This deterioration increases the demand in active control. However, what levels of demand in active control do older adults need? This study aimed to answer this question by using a novel assessment. Both young and old adults walked in three conditions: normal, slip, and slip with low light conditions. The amount of step length variability, step width variability, and the 95% confidence interval of the ellipse area of heel contact locations was measured to quantify and distinguish different levels of demand and patterns in active control. The results found that less sensory information led to a higher level of demand in active control in both anterior-posterior and medial-lateral directions. Importantly, different patterns in active control were found among different age groups and perturbation conditions. This study extended the current knowledge and further proposed the possibility of multiple patterns in active control. This study also suggests a new method to quantify the levels and patterns in active control under sensory perturbations, and this innovation can be used to guide age-related fall prevention training.

## Introduction

How sensory systems contribute to daily life activities has been well investigated in young and older adults (Lord et al., 1991; Kristinsdottir et al., 2001; Bacsi and Colebatch, 2005). The visual system conveys environmental information for defining self-position (Reimann et al., 2018). The somatosensory system, the only part of the body that directly contacts the ground while transferring the body from one point to another, predominantly uses the feedback loop mechanism to keep the balance (Rice and Albrecht, 2008). This feedback loop mechanism has been defined as a passive control (Bauby and Kuo, 2000). However, this capability of passive control is also deteriorated by aging (Dean et al., 2007). Therefore, for a human-walk, an active control, rather than only passive control, needs to be involved in dissolving these external perturbations to maintain the lateral balance (Bauby and Kuo, 2000; Dean et al., 2007; O’Connor and Kuo, 2009; Rebula et al., 2017).

These studies divide the control into two parts: active and passive control. Engagement in active control/passive control has been widely defined by gait variability. Higher gait variability infers active control; on the other hand, lower gait variability infers passive control (Bauby and Kuo, 2000; Dean et al., 2007; O’Connor and Kuo, 2009; Rebula et al., 2017). However, this hypothesis has its own fundamental flaws. For example, does active control have different levels of demand, or what is the minimum gait variability for passive control? These questions have not yet been answered. In the abovementioned studies, several observations have been used to explicitly quantify the level of demand in active control (1) the medial-lateral variability (step width variability) is larger than the anterior-posterior variability (step length variability) when walking normally (with intact visual, somatosensory, and vestibular systems) (Bauby and Kuo, 2000; Rebula et al., 2017); (2) the medial-lateral gait variability increases significantly when the visual information is absent, while the anterior-posterior gait variability is affected much less (Bauby and Kuo, 2000; Rebula et al., 2017); and (3) actively adjusting foot placement in the medial-lateral direction increases the step width variability and also increases the metabolic cost due to continuously redirecting the center of mass between steps (Dean et al., 2007). Based on these observations, higher gait variability seemingly links to higher demands in active control. The only study that clearly stated “great active control” is to link step width variability with lateral stabilization (Donelan et al., 2004). This study indicates that smaller step width variability supported by lateral stabilization requires less active control. However, the “levels of demand in active control” have still not been clearly defined. Moreover, a study further infers that old adults demand active control during walking due to the deterioration of sensory systems and sensory integration capabilities as people aged (Dean et al., 2007). Specifically, this demand in active control is speculated to attribute to the deterioration of the somatosensory system with age (Clark et al., 2014).

To verify the abovementioned questions, studies introduce a perturbation by manipulating the treadmill belt speeds or moving the platform of treadmill to induce accelerations/deceleration of speed for disturbing the somatosensory/proprioceptive systems during walking in young adults and old adults (Hak et al., 2012; McIntosh et al., 2017; Madehkhaksar et al., 2018; Roeles et al., 2018). Unsurprisingly, the gait variabilities increase significantly in both anterior-posterior and medial-lateral in both age groups (Madehkhaksar et al., 2018; Roeles et al., 2018). The step length and step width variabilities are particularly higher in old than in young adults, inferring those sensory perturbations might trigger the demands in active control in both anterior-posterior and medial-lateral directions (Madehkhaksar et al., 2018). However, the limitation of these previous studies is that by continuously exploring perturbations with the same amplitude and the same time interval, a learning effect may be triggered for participants who learn to expect perturbations, which may further hinder perturbations’ true impact on the outcome. In the real world, the perturbations might be given in a random fashion. For instance, the ice never accumulates equally over the ground. When stepping on such ground, an extra caution needs to be taken. Therefore, to understand the age-related changes in the levels of demand and patterns in active control, implementing the random fashions of perturbations is necessary.

In the current study, there were two novelties. Firstly, the continuous pseudo-random perturbations were introduced by manipulating the treadmill speeds. Secondly, a novel method, a 95% confidence interval of the ellipse area of heel contact locations, was used to measure the levels of demand and patterns in active control. By applying these two novelties, this study attempted to understand the important questions as follows: (1) did different levels of demand exist in active control? (2) did different patterns exist in active control? (3) did aging affect the levels or patterns in active control? We hypothesized that when perturbing multiple sensory systems during walking, humans required a higher level of demand in active control than normal walking conditions and walking under a single sensory system perturbed state. We also hypothesized that old adults require a higher level of demand in active control than young adults. Importantly, old adults might demonstrate different patterns in active control when receiving multiple sensory perturbed conditions in comparison with young adults.

## Materials and Methods

### Participants

**FIGURE 1:**
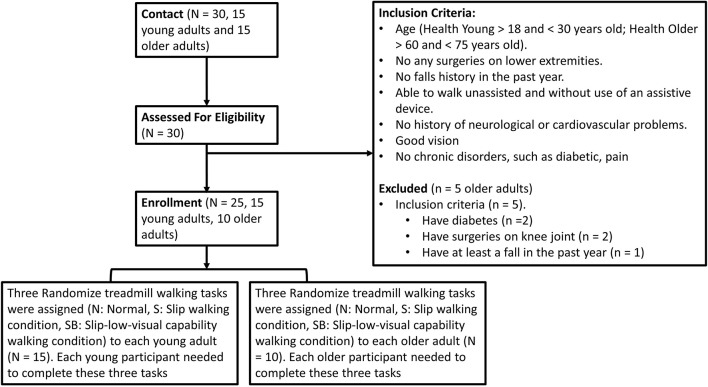
A consort diagram for the inclusion. The inclusion criteria were that the age for young adults should be between 18 and 30 years old. For older adults, the age range was set between 60 to 75 years old. In addition, participants were free from any musculoskeletal impairments, had no history of extremity injuries, and had no history of surgeries in their joints that may have affected their gait. They also had no falls in the past year. In addition, they could walk unassisted and without use of an assistive device. If they had any history of neurological disorders or chronic diseases, they were excluded from this study.

Fifteen young and 10 old adults (Young: 7 Males 8 Females, 22.53 ± 2.79 years old, Preferred Walking Speed: 1.47 ± 0.08 m/s, Height: 170.05 ± 7.21 cm, Weight: 64.47 ± 11.54 kg; Old: 6 Males 4 Females, 66.51 ± 4.55 years old, Preferred Walking Speed: 1.38 ± 0.09 m/s, Height: 172.70 ± 10.92 cm, Weight: 72.42 ± 22.07 kg) participated in this study. More detail is shown in Figure 1 and its caption. This study was carried out in accordance with the relevant guidelines and regulations of and upon approval by the University of Nebraska Medical Center Institutional Review Board (IRB# 340-10-FB). Informed consent was obtained from all participants before the experiments began.

### Experimental Setup

**FIGURE 2:**
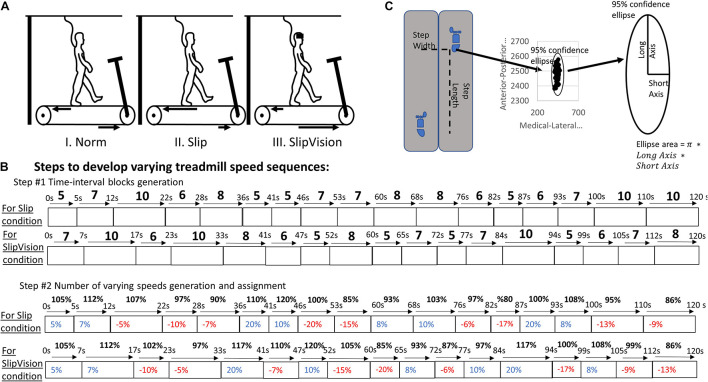
**(A)** Diagram for three locomotor tasks. **(B)** Steps to develop varying treadmill speed sequences: (1) time interval blocks—continuously and randomly generated a value (between 5 and 10) until the sum of these values reached 120 (in the current study, a total of seventeen time-interval blocks were generated); (2) % of PWS blocks—generated a number between –20 and 20 (positive value indicates acceleration, negative value indicates deceleration). Also, all added numbers from already generated numbers and the current generated number cannot reach –20 or 20; (3) assigned these numbers from the step #2 into time-internal blocks from the step #1 (in this figure). Five to ten seconds for a time interval was used to reduce adaptation of walking in the perturbed environment. These two pseudo-random varying speed scripts were applied to all participants to make the outcome consistent and interpretable. **(C)** The data processing method. For the levels of demand in active control, the step length variability and step width variability were used. For the patterns in active control, the 95% confidential interval of the ellipse area was calculated by each heel contact during the initial 200 steps.


Participants walked on an instrumental treadmill (Bertec Corp., Columbus, OH, United States). A motion capture system with 8 cameras (Optotak Certus; Northern Digital Inc., Waterloo, Canada) was used to capture the three-dimensional marker trajectories at a sampling rate of 100 Hz. Active rigid body markers were placed on the hip (greater trochanter), knee (lateral epicondyle), ankle (lateral malleolus), toe (2nd Metatarsal), and heel of each foot. The acceleration/deceleration of the treadmill speeds was controlled by our customized visual basic script (see scripts below, Microsoft, Redmond WA). Three walking conditions were randomly assigned to all participants as follows: normal walking condition (Norm), slip walking condition (Slip), and slip-low-visual capability condition (SlipVision). Each walking condition lasted for 2 min. We limited this 2-min timeframe to avoid adaptations. For Norm condition, participants ambulated on the treadmill at their preferred walking speed (PWS) without speed variation. Then the pseudo-random varying speeds in time and amplitude were calculated by the “Rnd” function in the visual basic program. More detail is shown in Figure 2. In order to measure the equal effect between these two pseudo-random varying speeds, the instant acceleration/deceleration was calculated using the follows equation:

$$\mathrm{The}\mathrm{instant}\mathrm{acceleration}\mathrm{or}\mathrm{deceleration}=\frac{\left|\mathrm{nth}\%\mathrm{of}\mathrm{PWS}\right|}{\mathrm{nth}\mathrm{of}\mathrm{time}\mathrm{interval}},\left(n=1ton=17\mathrm{in}\mathrm{the}\mathrm{current}\mathrm{study}\right)$$

In SlipVision condition, a reduced-light intensity goggle was made (MSA Safety Work, Pittsburgh, PA) with car tinting vinyl, where light intensity was decreased from 22 to 0.7 lx (150 lx is for regular office). The light intensity was measured by the light meter while inserting the light meter probe into the goggle (Dr. Meter, support@drmeter.com). Data processing was performed using a custom MATLAB code (Mathworks Inc., Natick, Massachusetts) for the calculation of step length, step width, step length variability, and step width variability using foot vertical velocity method (Chien et al., 2014); the 95% confidence interval of the ellipse area, the long axis of the ellipse, and the short axis of the ellipse (Figure 1). The normalized step length variability and normalized step width variability were defined as the coefficient of variation of each spatial gait parameter, because using the coefficient of variation was suggested as a more appropriate measure of gait variability than using the standard deviation (Wurdeman and Stergiou, 2013). Only toe and heel markers were used for defining the level of active control using the conventional spatial gait variability. A novel outcome measure, the 95% confidence interval of the ellipse area of heel contact locations, was proposed to measure not only the levels of demand in active control but also the patterns in active control. The higher area is associated with higher demands in active control (Bauby and Kuo, 2000; Schubert and Kirchner, 2014). Initial 200 steps of each participant were used for analysis and were considered enough for evaluating the means of step length, step width, and the gait variability of step length and step width based on the latest study (Owings and Grabiner, 2003). Two 95% confidence intervals of the ellipse areas were calculated by all heel contact locations in the left and right leg of the transverse plane (Figure 2).

### Experimental Protocol

Prior to data collection, each participant was instructed to walk on the treadmill to determine their PWS. First, participants stood on the foot rail without contacting the belt of the treadmill. Next, the experimenter started the treadmill and increased the belt velocity from 0 to 0.8 m/s (Chien et al., 2014). When the belt reached 0.8 m/s, participants were instructed to step onto the treadmill while holding the handrail. After participants began walking on the treadmill, the experimenter evaluated the participant’s PWS by asking, “Is this walking speed comfortable, like walking around the grocery store?” Based on the participant’s answers, the treadmill velocity was then increased or decreased in 0.1 m/s increments by the experimenter. The above procedure was performed repeatedly by the experimenter until the participant’s PWS was verified. Once a PWS was confirmed, the participants walked continuously for 5 min to familiarize themselves with the treadmill without holding the handrail. Participants still could hold the handrail if they felt unbalanced; however, they were not encouraged to do so if they did not feel unbalanced. After a familiarization period, participants were asked to take a mandatory rest for 2 min. Next, a total of three 2-min walking conditions (Norm, Slip, SlipVision) were assigned to each participant in randomized order. Each participant underwent each condition. Between walking conditions, a 2-min mandatory rest was given to participants for washing out the sensation.

### Statistical Analysis

The pair test was used to test the equal effect of two pseudo-random varying speeds. The Shapiro-Wilk Normality Test was used to test the normality of each dependent variable, with the alpha value set at 0.05. A two-way mixed ANOVA (3 with/without sensory-conflicted conditions × 2 age groups) was used to investigate the effect of different types of sensory conflicts, the effect of aging, and the interaction between these two effects on each dependent variable. The dependent variables were step length, normalized step length variability, step width, normalized step width variability, the 95% confidence interval of the ellipse area, the length of the long axis of the ellipse, and the length of the short axis of the ellipse. If an interaction existed in each dependent variable, *post-hoc* comparisons were performed using the Tukey method for comparing the effect of different types of sensory conflicts in each age group. In addition, the Tukey-Kramer test was performed in SPSS when the group sizes were unequal, which compared between age groups in each sensory-conflicted condition. The threshold of statistical significance level was set at 0.05. Also, the Welch’s *t*-test was used to compare the preferred walking speed between young and old adults. If the data was not normally distributed, the Brunner and Langer non-parametric longitudinal data model was used to investigate the within subject effect (3 with/without sensory-conflicted conditions) and the between subject effect (2 age groups) (Brunner et al., 2002). If an interaction existed in each dependent variable, *post-hoc* comparisons were performed using the Wilcoxon Signed Rank Test for comparing the effect of different types of sensory conflicts in each age group. Also, the Mann-Whitney *U*-test was used to compare between age groups in each sensory-conflicted condition. Statistical analysis was completed in SPSS 20.0 (IBM Corporation, Armond, NY). To understand the effect size, we used the partial eta squared method as this method has been widely used for measuring the effect size, based on Cohen’s guideline 0.138 for a large effect size, 0.059 for a moderate effect size, and 0.01 for a small effect size (Agrawal et al., 2009).

## Results

### The Equal Effect Between Two Pseudo-Random Varying Speeds

The pair *t*-test was greater than 0.05 in the instant acceleration/deceleration between two pseudo-random varying speeds, indicating no difference between these two sequences (1.72 ± ± ± 1.06 m/s^2^ for sequence #1 vs. 1.70 ± 0.91 m/s^2^ for sequence #2). In addition, the total amount of instance acceleration/decelerations changes between two different conditions were the same (−14% PWS for sequence #1 vs. −14% PWS for sequence #2, negative number means the % PWS of deceleration).

### Normality Tests

Shapiro-Wilk Test results were greater than 0.05 in all dependent variables and all age groups, indicating that the data were normally distributed.

### Preferred Walking Speeds

Using Welch’s *t*-test did not find a significant difference in preferred walking speed between young and old adults.

### The Effect of Aging

A significant effect of aging was found in the step length [*F*_(1,23)_ = 7.065, *p* = 0.014], the step length variability [*F*_(1,23)_ = 14.14, *p* = 0.001], step width variability [*F*_(1,23)_ = 8.502, *p* = 0.008], ellipse area [*F*_(1,23)_ = 12.11, *p* = 0.002], length of long axis of the ellipse [*F*_(1,23)_ = 8.97 *p* = 0.006], and length of short axis of the ellipse [*F*_(1,23)_ = 12.06, *p* = 0.002].

### The Effect of Sensory Perturbation (Table 1 and Figure 3)

A significant effect of Slip/SlipVision was found in the step length variability [*F*_(2, 46)_ = 77.69, *p* < 0.001], step width variability [*F*_(2, 46)_ = 13.37, *p* < 0.0001], ellipse area [*F*_(2, 46)_ = 11.23, *p* < 0.001], length of the long axis of the ellipse [*F*_(2, 46)_ = 5.86, *p* = 0.005], and length of the short axis of the ellipse [*F*_(2, 46)_ = 22.18, *p* < 0.001]. The *post hoc* marginal means indicated that providing both Slip/SlipVision increased step length variability (*p* < 0.001/*p* < 0.001, respectively), step width variability (*p* = 0.008/*p* < 0.001), ellipse area (*p* < 0.001/*p* < 0.001), and length of the short axis of the ellipse (*p* < 0.001/*p* < 0.001) in comparison with the normal walking condition. More detail is shown in Table 1 and Figure 2.

**TABLE 1:** Mean values of step length/step width and step length/step width variability in healthy young and old adults.

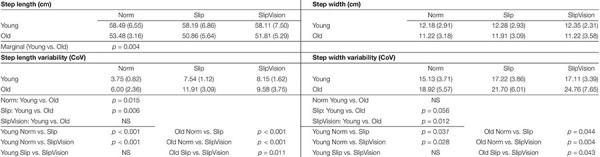

*The variability was defined by the coefficient of variation. NS, no significant, Means (Standard Deviation); Norm, normal walking condition; Slip, slip walking condition; SlipVision, slip with low light walking condition.*

**FIGURE 3:**
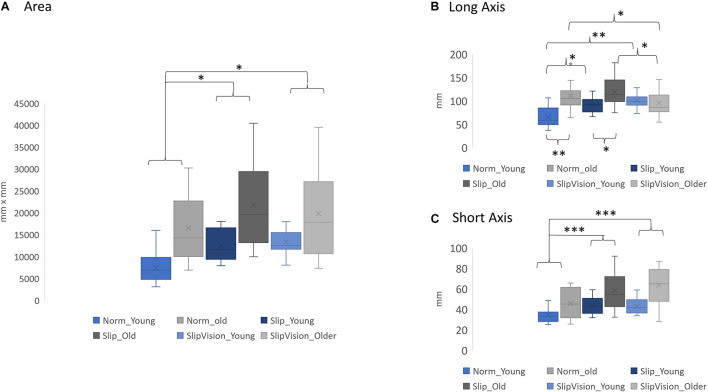
**(A)** Comparison of the 95% confidence interval of the ellipse area. **(B)** Comparison of the length of the long axis. **(C)** Comparison of the length of the short axis. ^∗^*p* < 0.05, ^∗∗^*p* < 0.01, ^∗∗∗^*p* < 0.001. Norm Young, normal walking in young adults; Norm Older, normal walking in older adults; Slip Young, pseudo-random varying treadmill walking in young adults; Slip Older, pseudo-random varying treadmill in older adults; SlipVision Young, pseudo-random varying treadmill walking with reduced-light intensity condition in young adults; and SlipVision Older, pseudo-random varying treadmill walking with reduced-light intensity condition in older adults. Higher gait variability indicated higher levels of demand in active control. On the other hand, lower gait variability indicated lower levels of demand in active control. Additionally, in the current study, a novel outcome measure, the 95% confidence interval of the ellipse area of heel contact locations, was proposed to measure not only the levels of active control but also the patterns in active control. A higher 95% confidence interval of the ellipse area indicated higher levels of demand in active control. The patterns in active control depended on the shapes of the ellipses, which was defined by the long axis and the short axis of the ellipse.

### The Interaction Between the Effect of Aging and the Effect of Perturbation

A significant interaction was found in the step length variability [*F*_(2, 46)_ = 8.186, *p* = 0.001], the step width variability [*F*_(2, 46)_ = 3.72, *p* = 0.032], and length of long axis [*F*_(2, 46)_ = 12.46, *p* < 0.001]. *Post hoc* comparisons indicated that providing Slip increased the length of the long axis of the ellipse in young adults (*p* = 0.002) in comparison with the Norm. Additionally, the length of the long axis of the ellipse increased in young adults (*p* < 0.001) but decreased in older adults (*p* = 0.024) when the SlipVision was given in comparison with the Norm (Figure 3). More detail is shown in Supplementary Material and Figure 3.

## Discussion

In the present study, we attempted to understand how randomly manipulating sensory systems affected the age-related changes in active control during walking. The results were in line with our hypotheses that (1) old adults used a different strategy than young adults to manage different perturbed conditions. (2) manipulating treadmill speeds randomly increased the demands in active control in both the anterior-posterior (AP) and medial-lateral (ML) directions during walking; and (3) with low visual support (SlipVision), the level of active control was demanded more in the medial-lateral direction in comparison with the full visual support condition (Slip).

### Aging Affected the Levels of Demand in Active Control

First, this study attempted to extend the definition of the levels of demand in active control. This study suggested that passive control was a low level of demand in active control. The direct observation was that both young and older adults still demonstrated a certain amount of gait variability in both AP and ML directions in Norm condition. If there was no active control involved, the gait variability should be zero, like robotic walking. Secondly, Dean et al. (2007) study supports the effect of aging directly, their study finds that step length variability is significantly larger in old than in young adults and there is no significant difference in step width variability between young and old adults; additionally, the step width variability in both groups is larger than the step length variability. In the present study, the same results were observed. These results suggested that in normal walking, old adults required a higher level of demand in active control in the AP direction than young adults. Also, both groups required a higher level of demand in active control in the ML direction than in the AP direction. Under perturbations in the current study, unsurprisingly, old adults required even a higher level of demands in active control than young adults. It might be due to the deterioration of sensory systems by aging. Specifically, under Slip/SlipVision conditions, the role of vestibular systems became crucial to maintain dynamic balance in the ML direction (Reimann et al., 2019), specifically for older adults (Bergström, 1973; Rosenhall and Rubin, 1975; Shaffer and Harrison, 2007; Agrawal et al., 2009). This was why the short axis (ML direction) had the largest increase (48.47%) in older adults than in young adults when walking under the SlipVision condition (both somatosensory and visual systems became unreliable simultaneously at this moment) than when walking under Slip (35.32% increase) and when walking in Norm (34.39% increase).

### The Patterns in Age-Related Active Control Were Different

Step length variability and width variability have been widely used for categorizing the active and passive control; however, these parameters could not assess the patterns in active control. In the current study, the 95% confidence interval of the ellipse area of heel contacts was used to assess the patterns in active control. From our observation (Figure 3), particularly for old adults, three different types of ellipses were observed as follows: (1) ellipse (area: 16517.38 mm^2^, long axis: 110.01 mm, short axis: 45.64 mm), (2) bigger ellipse (area: 21783.71 mm^2^, long axis: 119.28 short axis: 58.37), and (3) approach to the shape of a circle (area: 19382.15 mm^2^, long axis: 92.85 mm, short axis: 63.35 mm). However, for young adults, the types of ellipses were (1) ellipse (area: 7430.86 mm^2^, long axis: 64.34 mm, short axis: 33.96 mm), (2) bigger ellipse (area: 12494.28 mm^2^, long axis: 91.92, short axis: 43.14), and (3) biggest ellipse among conditions (area: 13399.34 mm^2^, long axis: 100.05, short axis: 42.67). This result clearly demonstrated that (1) old adults used different strategies than young adults to adapt the sensory conflicted conditions, particularly when both visual and somatosensory systems became unreliable, and (2) for old adults, there existed apparently different patterns in active control among different walking conditions. To our best knowledge, our study was the first study to clearly demonstrate this phenomenon. We speculated that the expanded areas of the ellipse of heel contacts during Slip and SlipVision conditions in young adults were to explore the environment and find an optimal foot placement to maintain balance. For old adults, when two sensory systems became unreliable, they had no choice but to limit their degrees of freedom in a small circle to walk conservatively, specifically they increased the length of the short axis (expand the control in ML direction) and decreased the length of the long axis (use minimum control in AP direction). These hypotheses were seemingly supported by Chien et al. (2014).

### The “Unexpected” Locomotor Tasks Increased the Levels of Demand in Active Control in Both Anterior-Posterior and Medial-Lateral Directions

O’Connor and Kuo (2009) further implement continuous visual perturbations in both AP and ML directions during walking on the treadmill and find that only visual perturbations in the ML direction significantly increases the step length variability and step width variability. O’Connor and Kuo’s (2009) finding suggests that (1) walking requires less control in the AP direction but requires more control in the ML direction; and (2) human respond to visual perturbation primarily in ML direction during walking. However, it is a different story when facing unexpected and perturbed environments. For the abovementioned second hypothesis, this present study was not in agreement. For O’Connor and Kuo’s (2009) study, when the rhythmic sinusoids visual perturbations are given, we speculated that young participants easily and quickly adapt a new walking strategy to this perturbing environment after a couple of steps, resulting in the only demand of active control in the ML direction. Madehkhaksar et al. (2018) agree with our speculation and indicate that when an unexpected perturbation in the forward, backward, left or right direction is randomly given to healthy young adults on the platform during walking, the active control requires significantly in both AP and ML directions no matter which direction the perturbation is given. This result aligns with our speculation that the locomotor tasks are the major contributors to the level of demand in active control and the major key point of these tasks is “unexpected.” In the present study, exploring continuous unexpected perturbations in the AP direction required participants to increase the levels of demand in active control in both AP and ML directions in both the young and old adults. The direct evidence was significantly larger step length variability and larger step width variability in perturbed conditions compared with normal walking conditions.

### Limitation and Future Application

The limitation of this study was the sample size of participants. Therefore, the effect size was calculated by using the Partial Eta Squared method. Partial Eta Squared values for the interaction between aging and different types of sensory-conflicted conditions were 0.226 in the step length variability, 0.135 in the step width variability, and 0.351 in the length of the long axis indicating a large effect size. Another potential limitation of this study was that the pattern in the mechanical perturbation sequence might not be counter-balanced within different conditions (Slip and SlipVision). This limitation might lead to failure to identify significant changes in the outcome variables within different conditions (Slip and SlipVision). To solve this limitation, the possible experimental design in future studies will be to assign half the young/older adults to sequence #1 with Slip and sequence #2 with SlipVision and assign another half to sequence #2 with Slip and sequence #1 with SlipVision. For this current study, two attempts were used to solve this issue of counter-balance within different conditions. The first attempt was to ensure these two sequences had the same effect on walking. Thus, the total amount of instance acceleration/deceleration changes between two sequences were set to be the same. The average of the instance acceleration/deceleration changes within two sequences was approximately the same. The second attempt was that the conditions of Norm, Slip, and SlipVision were assigned to participants in a random order to eliminate the learning effect from each sequence. A potential application of this work might be a new means to quantify dynamic stability during walking. The area of heel contacts (patterns in active control) might be used to identify different types of patients with different gait disorders and monitor their gait improvements after rehabilitation, such as patients with Parkinson’s and stroke survivors.

## Data Availability Statement

The original contributions presented in the study are included in the article/Supplementary Material, further inquiries can be directed to the corresponding author/s.

## Ethics Statement

The studies involving human participants were reviewed and approved by the University of Nebraska Medical Center Institutional Review Board (IRB# 340-10-FB). The patients/participants provided their written informed consent to participate in this study.

## Author Contributions

JC designed and carried out the experiments. JH and JC wrote the main text. JH analyzed the data. Both authors contributed to the article and approved the submitted version.

## Conflict of Interest

The authors declare that the research was conducted in the absence of any commercial or financial relationships that could be construed as a potential conflict of interest.

## Publisher’s Note

All claims expressed in this article are solely those of the authors and do not necessarily represent those of their affiliated organizations, or those of the publisher, the editors and the reviewers. Any product that may be evaluated in this article, or claim that may be made by its manufacturer, is not guaranteed or endorsed by the publisher.
