# Supplementary material for: Aging Affects the Demands and Patterns in Active Control Under Different Sensory-Conflicted Conditions
Source: Front Aging Neurosci. 2021 Nov 5;13:742035. doi: 10.3389/fnagi.2021.742035 (PMC8602863; doi:10.3389/fnagi.2021.742035)
Supplement: Supplementary file 1 [file Data_Sheet_1.docx]

|  | StepLength |  |  |  | StepLengthVariability |  |  |
| --- | --- | --- | --- | --- | --- | --- | --- |
| Young | Norm | Slip | SlipVision |  | Norm | Slip | SlipVision |
| Sub01 | 56.7988225 | 57.38915574 | 62.0511952 |  | 4.301192906 | 8.353615621 | 6.80932051 |
| Sub02 | 53.031992 | 49.72640826 | 59.0742122 |  | 2.39036355 | 8.22709856 | 6.933517911 |
| Sub03 | 55.7065815 | 57.63676946 | 55.5477999 |  | 3.90827903 | 5.986147568 | 7.936218129 |
| Sub04 | 61.9357654 | 56.68359186 | 61.0017849 |  | 2.750990337 | 6.899118552 | 5.839192988 |
| Sub05 | 58.5209657 | 57.24429925 | 62.851088 |  | 4.215991605 | 8.786237461 | 8.780906377 |
| Sub06 | 62.0235508 | 59.00443563 | 59.4434766 |  | 5.078827804 | 6.645461139 | 9.024162557 |
| Sub07 | 69.5527285 | 69.42205456 | 62.8600181 |  | 2.868147794 | 5.313787391 | 7.195544089 |
| Sub08 | 67.9404045 | 70.31969391 | 67.59293 |  | 4.593897722 | 6.569420265 | 7.058258512 |
| Sub09 | 59.6876068 | 61.4215212 | 64.6546627 |  | 2.404122774 | 7.892449643 | 7.06913577 |
| Sub10 | 53.6473514 | 51.82548039 | 55.9588785 |  | 4.202394627 | 9.000340489 | 12.52499947 |
| Sub11 | 62.8549667 | 61.58327802 | 55.1980859 |  | 3.780204744 | 7.218103304 | 8.028199185 |
| Sub12 | 65.7818797 | 67.59897191 | 66.3480119 |  | 3.394980327 | 8.683920017 | 7.577944893 |
| Sub13 | 51.3542196 | 51.57365224 | 44.40188 |  | 3.944161396 | 6.880623905 | 9.385004989 |
| Sub14 | 47.6686509 | 49.60337664 | 41.2030899 |  | 4.49965435 | 8.562898683 | 9.85318303 |
| Sub15 | 50.7832717 | 51.7468107 | 53.429877 |  | 3.867428006 | 8.03560676 | 8.176321255 |
| avg | 58.4859172 | 58.18529998 | 58.1077994 |  | 3.746709131 | 7.536988624 | 8.146127311 |
| std | 6.55185917 | 6.864550178 | 7.50046846 |  | 0.8196062 | 1.121420992 | 1.625804821 |
| Old | Norm | Slip | SlipVision |  | Norm | Slip | SlipVision |
| Sub01 | 54.8590678 | 58.47516965 | 58.8749265 |  | 9.482189095 | 10.60340609 | 11.21779199 |
| Sub02 | 48.6504063 | 44.57831099 | 50.0945283 |  | 5.988801701 | 10.64168976 | 9.096993726 |
| Sub03 | 49.6775085 | 51.12085046 | 49.2000603 |  | 4.982911877 | 9.922117779 | 9.722912659 |
| Sub04 | 54.84878 | 53.43370332 | 58.7511641 |  | 4.747248821 | 11.81849722 | 6.908143651 |
| Sub05 | 58.1662914 | 55.63000487 | 55.6068397 |  | 7.1584812 | 12.05200124 | 11.02325756 |
| Sub06 | 53.4842688 | 52.57322706 | 55.1779237 |  | 4.746340475 | 12.31670555 | 8.132848318 |
| Sub07 | 56.1835556 | 52.62534874 | 48.6744295 |  | 4.94404317 | 10.17437948 | 8.893366941 |
| Sub08 | 54.7138398 | 51.4794 | 45.9113506 |  | 2.727578191 | 10.65961413 | 3.856631167 |
| Sub09 | 54.848112 | 50.28995752 | 52.547734 |  | 4.807129399 | 9.296639142 | 8.558699991 |
| Sub10 | 49.4676234 | 38.47736 | 43.2162909 |  | 10.42152301 | 13.49108297 | 18.43151548 |
| avg | 53.4899454 | 50.86833326 | 51.8055248 |  | 6.000624694 | 11.09761334 | 9.584216148 |
| std | 3.16793186 | 5.646873773 | 5.2980943 |  | 2.36736967 | 1.280929419 | 3.754593578 |

Supplementary data #1: These were the composed data for step length and step length variability from 15 healthy young and 10 older adults. Normal walking condition (Norm), slip walking condition (Slip), and slip-low-visual capability condition (SlipVision). Avg: average values, Std: standard deviation.

Supplementary data #2: These were the composed data for step width and step width variability from 15 healthy young and 10 older adults. Normal walking condition (Norm), slip walking condition (Slip), and slip-low-visual capability condition (SlipVision). Avg: average values, Std: standard deviation.

|  | StepWidth |  |  |  | StepWidthVariability |  |  |
| --- | --- | --- | --- | --- | --- | --- | --- |
| Young | Norm | Slip | SlipVision |  | Norm | Slip | SlipVision |
| Sub01 | 16.57176985 | 16.88560021 | 14.87837796 |  | 14.21651481 | 12.01167355 | 20.02370946 |
| Sub02 | 12.25818667 | 13.30810907 | 12.69104623 |  | 13.40276844 | 10.73459603 | 15.10631457 |
| Sub03 | 12.43779354 | 10.98276144 | 11.97202256 |  | 12.88317249 | 16.15864916 | 18.79722759 |
| Sub04 | 11.33637711 | 12.51836534 | 11.70083527 |  | 20.48116552 | 20.08130135 | 21.35111076 |
| Sub05 | 16.56121173 | 17.01695748 | 16.60155409 |  | 11.39651354 | 17.66003409 | 16.27685004 |
| Sub06 | 13.86300442 | 12.90185149 | 12.69201561 |  | 15.32971846 | 17.76636872 | 18.91409122 |
| Sub07 | 9.897977208 | 9.918042703 | 9.793039 |  | 17.0902792 | 22.51207986 | 21.366549 |
| Sub08 | 12.7016576 | 12.6393833 | 13.6828102 |  | 14.14359136 | 17.72971419 | 13.95678359 |
| Sub09 | 7.046974604 | 7.086750777 | 8.200387368 |  | 19.25870213 | 23.99386147 | 22.67473637 |
| Sub10 | 10.90460881 | 12.50914459 | 10.72944773 |  | 14.72959999 | 17.258537 | 14.9234659 |
| Sub11 | 6.958090713 | 7.99229428 | 9.537638637 |  | 24.02425171 | 19.48208912 | 17.61600393 |
| Sub12 | 15.02268365 | 15.69015412 | 14.04367797 |  | 13.05408746 | 14.71874207 | 16.77906267 |
| Sub13 | 10.49618745 | 10.24031465 | 11.07992541 |  | 14.75230315 | 17.83748286 | 14.20376159 |
| Sub14 | 14.37551572 | 14.09905748 | 15.32042 |  | 10.08621617 | 10.99550709 | 10.47355031 |
| Sub15 | 12.33033999 | 10.45357765 | 12.27895539 |  | 12.04239 | 19.41287506 | 14.32217448 |
| avg | 12.1841586 | 12.28282431 | 12.34681023 |  | 15.12608496 | 17.22356744 | 17.1190261 |
| std | 2.910336302 | 2.932363419 | 2.309068833 |  | 3.703614442 | 3.858689975 | 3.398742411 |
| Old | Norm | Slip | SlipVision |  | Norm | Slip | SlipVision |
| Sub01 | 8.012880077 | 9.424437535 | 8.545519377 |  | 27.49622634 | 27.6676106 | 35.56319212 |
| Sub02 | 13.88783359 | 12.83063315 | 10.53367949 |  | 14.53399231 | 24.70027029 | 31.63876045 |
| Sub03 | 10.55772596 | 10.3723274 | 10.59300346 |  | 24.63113539 | 26.81428922 | 32.8401075 |
| Sub04 | 11.52869758 | 13.54050405 | 10.62739016 |  | 18.21857486 | 22.18446497 | 19.38444295 |
| Sub05 | 4.258310906 | 4.978170013 | 3.729082524 |  | 21.43239233 | 29.64125841 | 27.57224019 |
| Sub06 | 14.76153366 | 14.19518077 | 14.69455048 |  | 12.99330581 | 14.93834957 | 15.65631853 |
| Sub07 | 11.50469506 | 10.69541464 | 11.15790799 |  | 12.32472339 | 14.87663869 | 14.28086394 |
| Sub08 | 10.47050722 | 14.54766412 | 12.28116648 |  | 25.43717494 | 24.41914317 | 26.51515395 |
| Sub09 | 13.84009137 | 13.52183471 | 12.9705933 |  | 13.81352687 | 12.41553298 | 17.07603698 |
| Sub10 | 13.39173813 | 14.99466811 | 17.07826885 |  | 18.35226382 | 19.36213378 | 27.07497942 |
| avg | 11.22140136 | 11.91008345 | 11.22111621 |  | 18.92333161 | 21.70196917 | 24.7602096 |
| std | 3.182740389 | 3.088807083 | 3.575663843 |  | 5.574795757 | 6.013014284 | 7.647018607 |

Supplementary data #3: These were the composed data for long axis, short axis and area of 95% confidence interval ellipse from 15 healthy young and 10 older adults. Normal walking condition (Norm), slip walking condition (Slip), and slip-low-visual capability condition (SlipVision). Avg: average values, Std: standard deviation.

|  | Long-axis |  |  | Short-axis | |  | Area |  |  |
| --- | --- | --- | --- | --- | --- | --- | --- | --- | --- |
| Young | Norm | Slip | SlipVision | Norm | Slip | SlipVisioNorm | Norm | Slip | SlipVisioNorm |
| Sub01 | 58.3233 | 65.9235 | 77.0790 | 44.5452 | 45.3634 | 49.1615 | 8183.0032 | 9390.2139 | 11898.4726 |
| Sub02 | 48.7938 | 74.2859 | 72.8059 | 25.1996 | 37.1915 | 35.3504 | 3836.7085 | 8675.2063 | 8081.4772 |
| Sub03 | 36.5667 | 70.8418 | 100.3134 | 26.9073 | 35.7649 | 38.9803 | 3114.8373 | 7955.6562 | 12278.1617 |
| Sub04 | 48.5008 | 75.8466 | 98.7755 | 34.1952 | 42.9225 | 40.3415 | 5212.7556 | 10222.3548 | 12512.1208 |
| Sub05 | 106.0385 | 92.9271 | 98.6512 | 48.1636 | 58.8548 | 50.1620 | 16016.7411 | 17173.3114 | 15538.4288 |
| Sub06 | 84.3764 | 86.8246 | 90.5725 | 37.1378 | 50.5028 | 50.1184 | 9845.9134 | 13768.5383 | 14253.5589 |
| Sub07 | 92.6587 | 101.0973 | 114.9343 | 37.2791 | 52.9183 | 46.7282 | 13612.4174 | 16798.6775 | 16863.8970 |
| Sub08 | 86.3534 | 91.6066 | 107.8854 | 41.5377 | 45.9886 | 43.1349 | 11285.2732 | 13228.3722 | 14612.3989 |
| Sub09 | 39.6384 | 91.2007 | 108.3857 | 25.2597 | 32.8475 | 33.5262 | 3141.3153 | 9406.5557 | 11410.0242 |
| Sub10 | 52.0434 | 102.6900 | 87.5682 | 29.2939 | 51.4311 | 34.8067 | 4819.1205 | 16583.7718 | 9570.6055 |
| Sub11 | 67.5783 | 103.9073 | 103.0126 | 27.8215 | 32.6392 | 36.1349 | 6931.1340 | 10649.1563 | 11688.1881 |
| Sub12 | 61.3577 | 119.8384 | 97.7699 | 37.1899 | 47.9798 | 58.7058 | 7181.7060 | 18054.4242 | 18022.5494 |
| Sub13 | 55.0827 | 94.9070 | 97.1784 | 30.1813 | 31.6918 | 39.1518 | 5221.1367 | 9444.4029 | 11946.7745 |
| Sub14 | 76.8192 | 120.6070 | 118.0082 | 33.1492 | 38.1571 | 41.5062 | 8005.1780 | 14450.3019 | 15379.9337 |
| Sub15 | 50.8961 | 86.3316 | 127.8286 | 31.5961 | 42.8402 | 42.1880 | 5055.6890 | 11613.1855 | 16933.5043 |
| avg | 64.3352 | 91.9224 | 100.0513 | 33.9638 | 43.1396 | 42.6665 | 7430.8619 | 12494.2753 | 13399.3397 |
| std | 20.5805 | 16.2645 | 14.6091 | 6.9947 | 8.2981 | 7.0827 | 3836.2650 | 3454.2610 | 2838.4884 |
| Old | Norm | Slip | SlipVisioNorm | Norm | Slip | SlipVisioNorm | Norm | Slip | SlipVisioNorm |
| Sub01 | 178.6862 | 111.5849 | 145.3236 | 53.8366 | 60.2956 | 86.5627 | 30235.6650 | 21032.4629 | 39528.3984 |
| Sub02 | 91.8947 | 93.2303 | 97.7325 | 36.4769 | 48.5813 | 76.8595 | 10455.2791 | 11274.9698 | 23631.9234 |
| Sub03 | 100.9858 | 154.2273 | 83.5829 | 65.5134 | 65.1474 | 66.8850 | 20764.3311 | 31562.3000 | 17563.1746 |
| Sub04 | 63.6199 | 120.0236 | 86.7987 | 43.8942 | 62.8118 | 48.5272 | 8797.5654 | 24551.1312 | 13288.1225 |
| Sub05 | 143.5010 | 141.1491 | 142.2091 | 60.7752 | 91.2871 | 84.4347 | 27429.6363 | 40459.1905 | 37643.0325 |
| Sub06 | 110.2720 | 115.5599 | 74.9479 | 45.8114 | 48.5325 | 45.4340 | 15867.3290 | 18082.7965 | 10732.9553 |
| Sub07 | 114.0959 | 181.2566 | 84.9011 | 30.0615 | 32.1896 | 27.6366 | 10739.1579 | 18295.0289 | 7375.4584 |
| Sub08 | 87.3767 | 74.3901 | 54.2994 | 25.3513 | 43.3930 | 62.5789 | 6955.4478 | 9996.4750 | 10669.7205 |
| Sub09 | 102.0668 | 101.5115 | 81.8515 | 32.0379 | 39.8810 | 59.1140 | 12750.9430 | 13837.7577 | 15193.1145 |
| Sub10 | 107.6299 | 99.8727 | 76.8053 | 62.6660 | 91.6612 | 75.4475 | 21178.4614 | 28744.9801 | 18195.5701 |
| avg | 110.0129 | 119.2806 | 92.8452 | 45.6424 | 58.3780 | 63.3480 | 16517.3816 | 21783.7092 | 19382.1470 |
| std | 31.6184 | 31.6234 | 29.0294 | 14.5582 | 20.2906 | 18.7363 | 8047.1014 | 9657.7833 | 11113.9012 |

Supplementary data #4: These were the data for two sequences of instant changes in time and speed of the treadmill. S1: sequence #1, S2: sequence #2, A/D: percentage of increase/decrease from the preferring walking speeds. Time: how many seconds for keeping the same speed. abs(A/D) / time: the changes of the treadmill speed within certain time.

| S1 |  |  |  |  | S2 |  |  |
| --- | --- | --- | --- | --- | --- | --- | --- |
| A/D | Time | abs(A/D) / time |  |  | A/D | Time | abs(A/D) / time |
| 5 | 5 | 1 |  |  | 5 | 5 | 1 |
| 7 | 7 | 1 |  |  | 7 | 10 | 0.7 |
| -5 | 10 | 0.5 |  |  | -10 | 6 | 1.66666667 |
| -10 | 6 | 1.66666667 |  |  | -5 | 10 | 0.5 |
| -7 | 8 | 0.875 |  |  | 20 | 8 | 2.5 |
| 20 | 5 | 4 |  |  | -7 | 6 | 1.16666667 |
| 10 | 5 | 2 |  |  | 10 | 5 | 2 |
| -20 | 7 | 2.85714286 |  |  | -15 | 8 | 1.875 |
| -15 | 7 | 2.14285714 |  |  | -20 | 5 | 4 |
| 8 | 8 | 1 |  |  | 8 | 7 | 1.14285714 |
| 10 | 8 | 1.25 |  |  | -6 | 5 | 1.2 |
| -6 | 6 | 1 |  |  | 10 | 7 | 1.42857143 |
| -17 | 5 | 3.4 |  |  | 20 | 10 | 2 |
| 20 | 6 | 3.33333333 |  |  | -17 | 5 | 3.4 |
| 8 | 7 | 1.14285714 |  |  | 8 | 6 | 1.33333333 |
| -13 | 10 | 1.3 |  |  | -9 | 7 | 1.28571429 |
| -9 | 10 | 0.9 |  |  | -13 | 8 | 1.625 |
| -14 |  |  |  |  | -14 |  |  |
|  | AVG. | 1.72752101 |  |  |  | AVG. | 1.69551821 |
|  | STD | 1.05636902 |  |  |  | STD | 0.90800684 |
