# Supplementary figures and images for: Aging Affects the Demands and Patterns in Active Control Under Different Sensory-Conflicted Conditions
Source: Front Aging Neurosci. 2021 Nov 5;13:742035. doi: 10.3389/fnagi.2021.742035 (PMC8602863; doi:10.3389/fnagi.2021.742035)

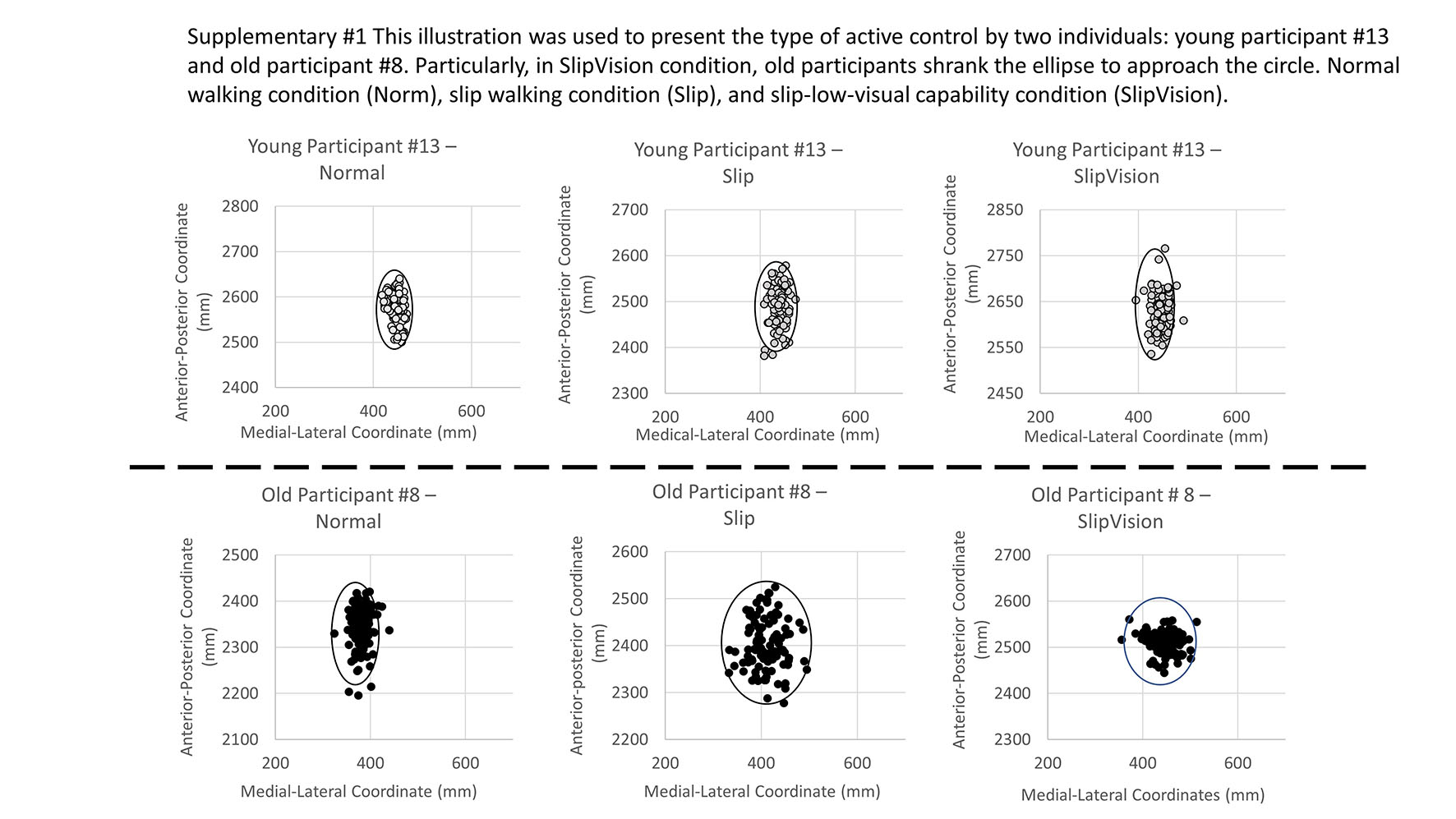

Supplement: Supplementary file 2 [file Image_1.jpg]
